# Annexin A10 in Human Oral Cancer: Biomarker for Tumoral Growth via G1/S Transition by Targeting MAPK Signaling Pathways

**DOI:** 10.1371/journal.pone.0045510

**Published:** 2012-09-17

**Authors:** Toshihiro Shimizu, Atsushi Kasamatsu, Ayumi Yamamoto, Kazuyuki Koike, Shunsaku Ishige, Hiroaki Takatori, Yosuke Sakamoto, Katsunori Ogawara, Masashi Shiiba, Hideki Tanzawa, Katsuhiro Uzawa

**Affiliations:** 1 Department of Clinical Molecular Biology, Graduate School of Medicine, Chiba University, Inohana, Chuo-ku, Chiba, Japan; 2 Division of Dentistry and Oral-Maxillofacial Surgery, Chiba University Hospital, Chuo-ku, Chiba, Japan; 3 Department of Molecular Genetics, Graduate School of Medicine, Chiba University, Chuo-ku, Chiba, Japan; University of Pittsburgh School of Medicine, United States of America

## Abstract

**Background:**

Annexins are calcium and phospholipid binding proteins that form an evolutionary conserved multigene family. Considerable evidence indicates that annexin A10 (ANXA10) is involved in tumoral progression, although little is known about its role in human oral carcinogenesis. In this study, we investigated the involvement of ANXA10 in oral squamous cell carcinoma (OSCC).

**Methodology/Principal Findings:**

ANXA10 mRNA and protein expressions were assessed by quantitative reverse transcriptase polymerase chain reaction and immunoblotting, and we conducted a proliferation assay and cell-cycle analysis in ANXA10 knockdown cells *in vitro*. We evaluated the correlation between the ANXA10 expression status in 100 primary OSCCs and the clinicopathological features by immunohistochemistry. ANXA10 mRNA and protein expression levels were up-regulated in all cellular lines examined (n = 7, *p*<0.05). ANXA10 knockdown cells showed that cellular proliferation decreased by inactivation of extracellular regulated kinase (ERK) (*p*<0.05), and cell-cycle arrest at the G1 phase resulted from up-regulation of cyclin-dependent kinase inhibitors. ANXA10 protein expression in primary OSCCs was also significantly greater than in normal counterparts (*p*<0.05), and higher expression was correlated with tumoral size (*p* = 0.027).

**Conclusions/Significance:**

Our results proposed for the first time that ANXA10 is an indicator of cellular proliferation in OSCCs. Our results suggested that ANXA10 expression might indicate cellular proliferation and ANXA10 might be a potential therapeutic target for the development of new treatments for OSCCs.

## Introduction

Annexins are calcium and phospholipid binding proteins that form an evolutionary conserved multigene family. Dysregulation of annexin family members has been reported in numerous cancers and affects patterns of cellular behaviors, such as proliferation, invasiveness, and cancer-related signaling pathways, suggesting that annexins may play important roles in tumoral development and progression [1]–[10]. Annexin A10 (ANXA10), an overexpressed gene in the oral squamous cell carcinomas (OSCC)-derived cellular lines in our previous microarray data [11], has been implicated in cellular function in endocytosis and exocytosis; anticoagulant activity; interaction with the cytoskeleton; differentiation; and cellular proliferation [12], [13]; and the relevance of malignancy in Barrett's esophagus, gastric cancer, and bladder cancer [14]–[16].

Proliferation of human cancer cells is largely controlled in the G1 phase of the cellular cycle. The mitogen-activated protein kinase (MAPK) signaling cascades have emerged as major players in proliferation in various cancer cells [17]. Previous studies have reported that several annexins specifically modulate the extracellular regulated kinase (ERK)/MAPK signaling cascade upstream [18], [19]. ERK activation plays a fundamental role in G1/S transition [20], [21]. Members of the cyclin*-*dependent kinase (CDK) interacting protein/kinase inhibitory protein (Cip/Kip) family bind to cyclin-CDK complexes and inhibit their activities, leading to G1 cell-cycle arrest. Cyclin D1, cyclin E, p21^Kip1^, and p27^Kip1^ levels are affected by multiple signaling pathways including the ERK/MAPK signaling pathway [22]–[25]. However, the molecular mechanisms by which ANXA10 modulates these cellular responses have not been fully elucidated in OSCCs.

We report the results of a comprehensive analysis of aberrant expression of ANXA10 in OSCCs that are functionally and clinically linked to tumoral progression.

## Results

### Evaluation of ANXA10 mRNA and protein expression in OSCC-derived cellular lines

**Figure 1:**
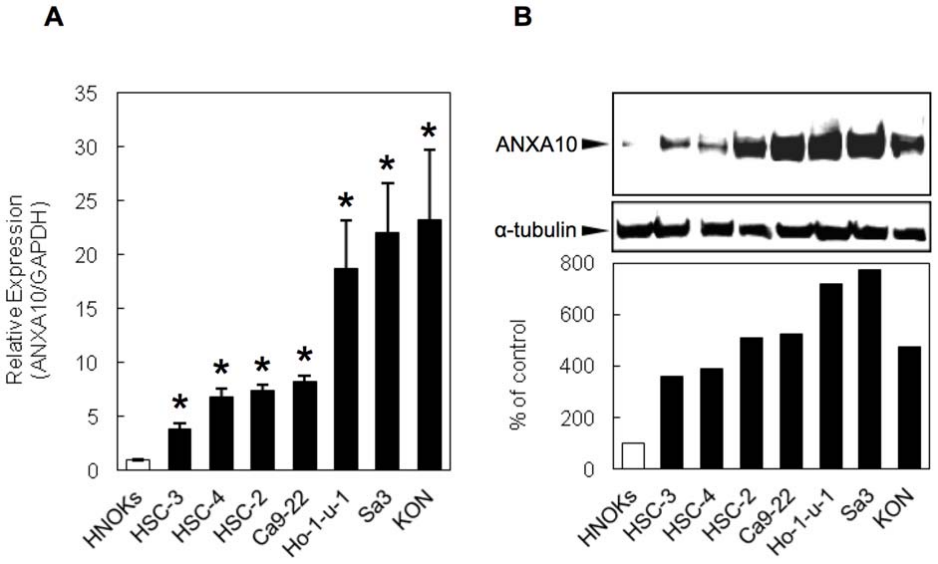
Evaluation of ANXA10 expression in OSCC-derived cellular lines. (A) Quantification of *ANXA10* mRNA levels in OSCC-derived cellular lines by qRT-PCR analysis. Significant up-regulation of *ANXA10* mRNA is seen in the seven OSCC-derived cellular lines compared with that in the HNOKs. Data are expressed as the means ± SEM of values from three assays (*p<0.05; Mann-Whitney U test). (B) Immunoblotting analysis of ANXA10 protein in the OSCC-derived cellular lines and HNOKs. ANXA10 protein expression (molecular weight, 37 kDa) is up-regulated in the OSCC-derived cellular lines compared with that in the HNOKs. Densitometric ANXA10 protein data are normalized to α-tubulin protein levels. The values are expressed as a percentage of the HNOKs.

To investigate the expression status of ANXA10 identified as a cancer-related gene by our previous microarray data [11], we performed quantitative real-time reverse transcription-PCR (qRT-PCR) and immunoblotting analyses using seven OSCC-derived cellular lines and human normal oral keratinocytes (HNOKs). ANXA10 mRNA and protein expression status were significantly up-regulated in all OSCC cellular lines compared with that in the HNOKs (Figure 1A, B; *p<0.05). Expression analysis indicated that both transcription and translation products of this molecule were highly expressed in OSCC-derived cellular lines.

### Establishment of ANXA10 knockdown cells

**Figure 2:**
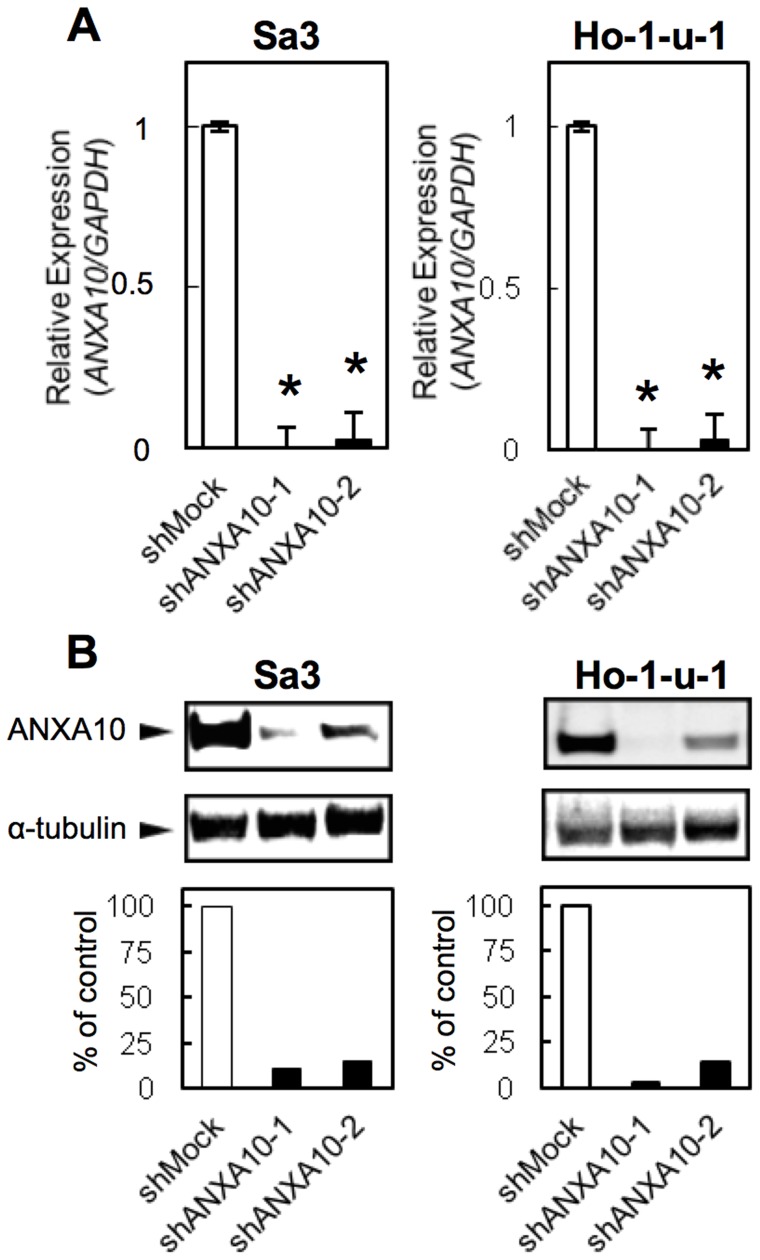
Expression of ANXA10 in shANXA10-transfected cells. (A) qRT-PCR shows that ANXA10 mRNA expression in the shANXA10-transfected cells (Sa3- and Ho-1-u-1-derived transfectants; 2 clones each) are significantly lower than in the shMock-transfected cells (*p<0.05; Mann-Whitney U test). (B) Immunoblotting analysis shows that the ANXA10 protein levels in shANXA10-transfected cells (Sa3- and Ho-1-u-1-derived transfectants; 2 clones each) also have decreased markedly compared with that in the shMock-transfected cells.

Since ANXA10 expression was up-regulated in the OSCC cellular lines, we assumed that ANXA10 might play an important role in OSCCs. To assess the ANXA10 functions *in vitro*, an shRNA experiment was carried out using the Sa3 and Ho-1-u-1 cells. Expressions of ANXA10 mRNA and protein in the shANXA10-transfected cells were significantly lower than in the shMock-transfected cells (Sa3 and Ho-1-u-1-derived transfectant cells, Figure 2A, B; *p<0.05).

### Functional analyses of ANXA10 knockdown cells

**Figure 3:**
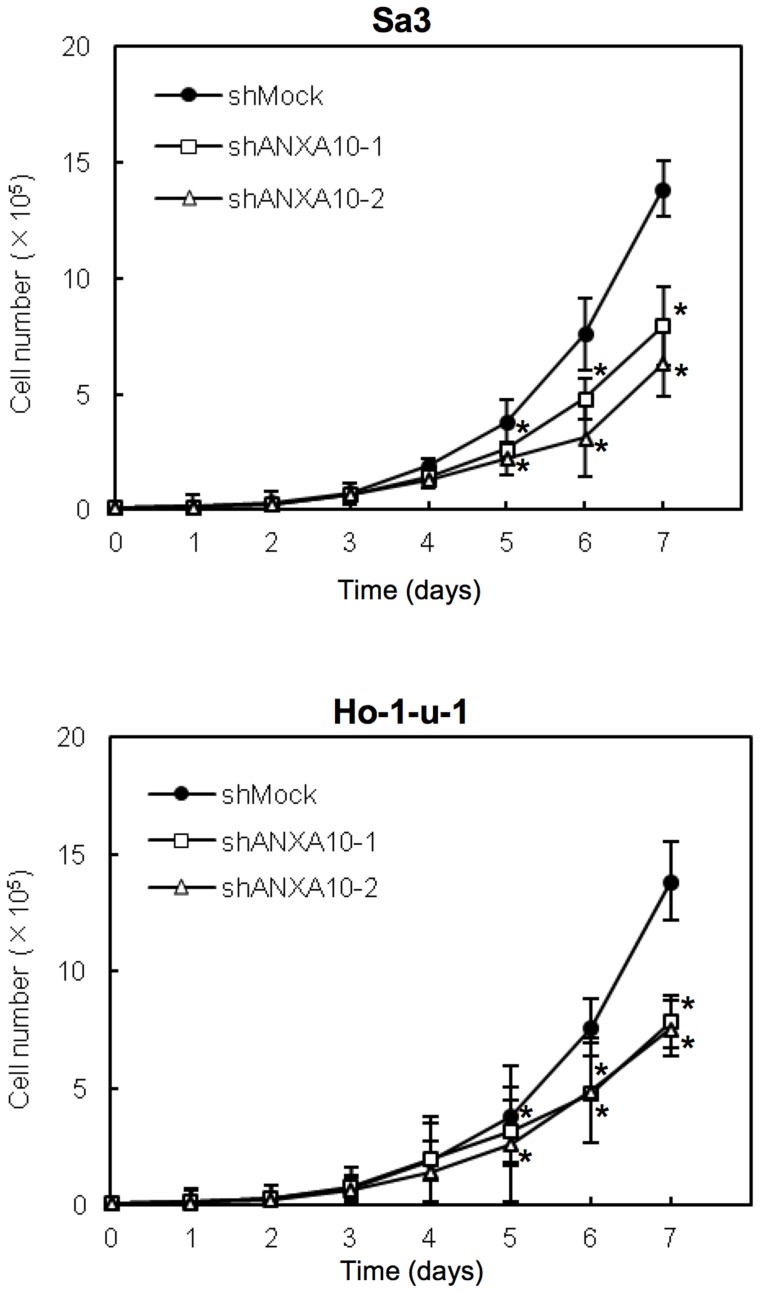
Effect of ANXA10 knockdown in shANXA10-transfected cells. To determine the effect of shANXA10 on cellular proliferation, shANXA10- and shMock-transfected cells are seeded in 6-well plates at a density of 1×10^4^ viable cells/well. Both transfectants were counted on 7 consecutive days. The cellular growth of shANXA10-transfected cells (Sa3- and Ho-1-u-1-derived transfectants; 2 clones each) is significantly inhibited compared with shMock-transfected cells after 7 days (168 hours). The results are expressed as the means ± SEM of values from three assays. The asterisks indicate significant differences between the shANXA10- and shMock-transfected cells (*p<0.05; Mann-Whitney U test).

To evaluate the effect of ANXA10 knockdown on cellular growth, we performed a cellular proliferation assay. Transfected with the ANXA10 shRNA (shANXA10)- and the control shRNA (shMock)-transfected cells were seeded in six-well plates at a density of 1×10^4^ viable cells/well counted on 7 consecutive days. There was a significant decrease in cellular growth of the shANXA10-transfected cells compared with the shMock-transfected cells (Sa3 or Ho-1-u-1-derived transfectant cells, Figure 3; *p<0.05).

**Figure 4:**
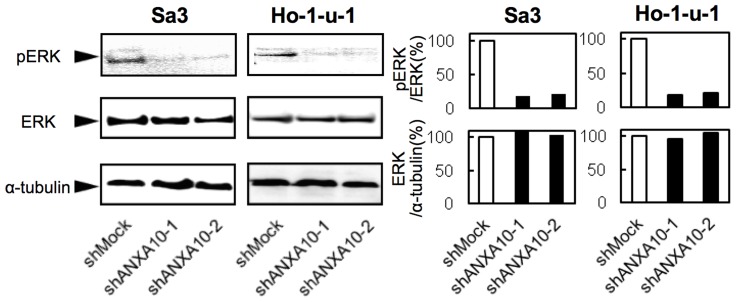
ANXA10 knockdown inhibits ERK activation. ANXA10 knockdown causes decreased levels of phosphorylated ERK (pERK) compared with the shMock-transfected cells (Sa3- and Ho-1-u-1-derived transfectants; 2 clones each); the ERK level is unchanged. Densitometric pERK/ERK protein data are normalized to α-tubulin protein levels.

To investigate a potential underlying mechanism that would explain reduced cellular proliferation in the shANXA10-transfected cells, we assessed the ERK pathways, which are frequently up-regulated in endometrial and other cancers [26]–[29]. Phosphorylated ERK (pERK) protein decreased significantly in shANXA10-transfected cells compared with shMock-transfected cells (Figure 4). These results suggested that the ERK signaling pathway is frequently attenuated in shANXA10-transfected cells.

**Figure 5:**
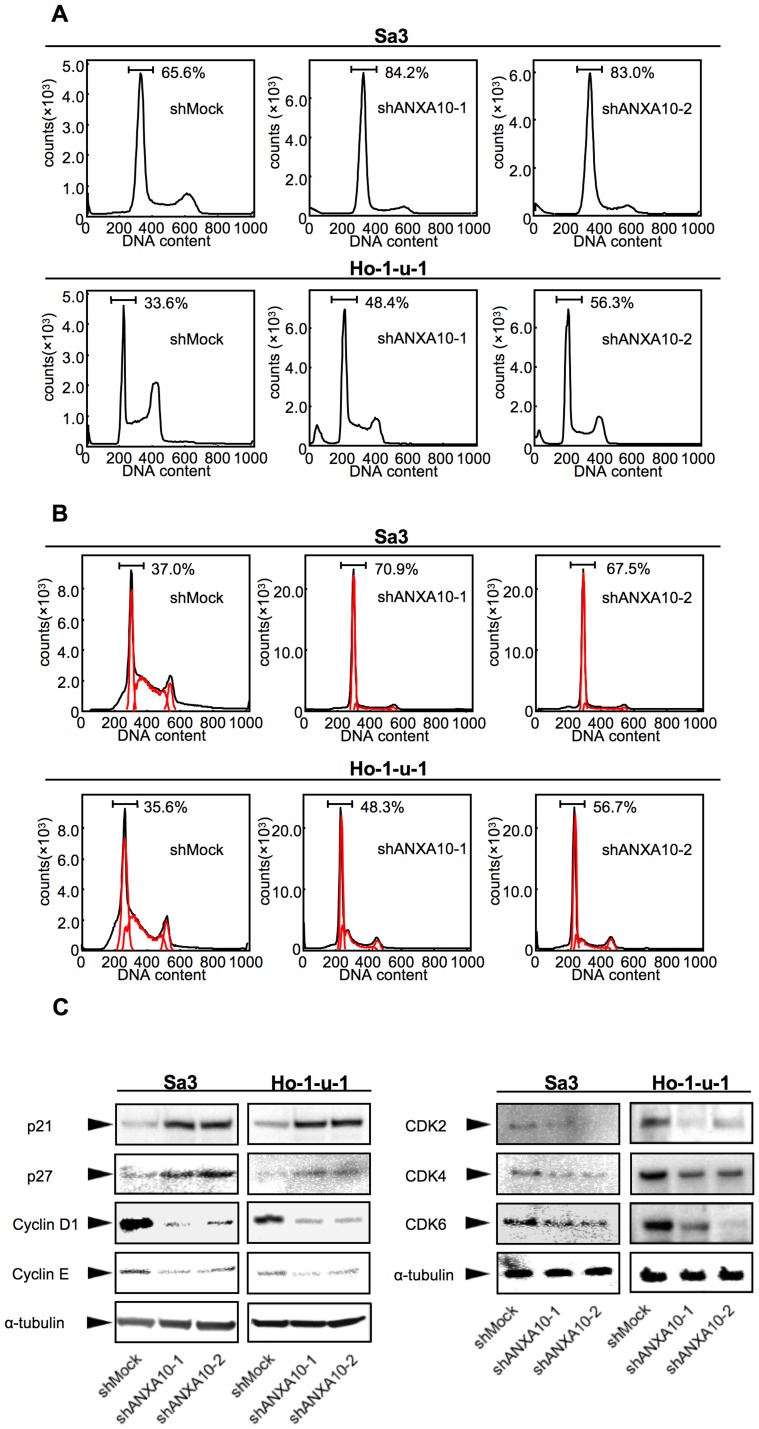
shANXA10 promotes G1 arrest. To investigate cell-cycle progression, we analyzed flow cytometric determination of DNA content by a FACScalibur in the G0–G1, S, and, G2–M phases. We determined the expression level of CDKIs (p21^Cip1^and p27^Kip1^), cyclin D1, cyclin E, CDK2, CDK4, and CDK6 to identify the mechanism by which ANXA10 inhibits cell-cycle progression in the G1 phase. (A) After synchronizing at G0/G1 phase with deprived of serum, flow cytometric analysis was performed to investigate the cell cycle in the shANXA10- and shMock-transfected cells. The percentage of the G1 phase in the shANXA10-transfected cells (Sa3- and Ho-1-u-1-derived transfectants; 2 clones each) has increased markedly compared with the mock-transfected cells (*p*<0.05, Mann-Whitney's *U* test). (B) After synchronizing at G2/M phase to treated with nocodazol, flow cytometric analysis was performed to investigate the cell cycle in the shANXA10- and shMock-transfected cells. The percentage of the G1 phase in the shANXA10-transfected cells (Sa3- and Ho-1-u-1-derived transfectants; 2 clones each) has also increased markedly compared with the mock-transfected cells (*p*<0.05, Mann-Whitney's *U* test). (C) Immunoblotting analysis shows up-regulation of p21^Cip1^and p27^Kip1^ and down-regulation of cyclin D1, cyclin E, CDK2, CDK4, and CDK6 in the shANXA10-transfected cells (Sa3- and Ho-1-u-1-derived transfectants; 2 clones each) compared with the shMock-transfected cells.

To investigate the mechanism of cell-cycle progression in shANXA10-transfected cells, we performed flow cytometric analysis of shANXA10-transfected cells. The percentage of the G1 phase in shANXA10-transfected cells was significantly (*p*<0.05) higher than that in mock-transfected cells (Figure 5A, B). We also assessed the expression level of cyclin-dependent kinase inhibitors (CDKIs: p21^Cip1^ and p27^Kip1^), cyclin D1, cyclin E, CDK2, CDK4, and CDK6. As expected, while the CDKIs were up-regulated, a significant down-regulation of cyclin D1, cyclin E, CDK2, CDK4, and CDK6 were detected in shANXA10-transfected cells (Figure 5C). These results indicated that down-regulation of ANXA10 inhibited cellular proliferation by arresting the G1 phase.

### Evaluation of ANXA10 expression in primary OSCCs

**Figure 6:**
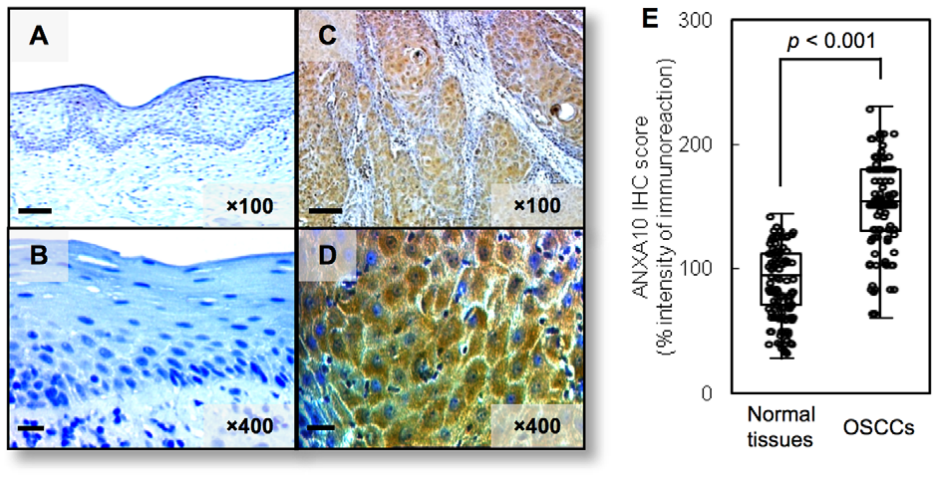
Evaluation of ANXA10 protein expression in primary OSCCs. Representative IHC results for ANXA10 protein in normal oral tissue (A, B) and primary OSCC (C, D) (A, C) Original magnification, ×100. Scale bars, 50 μm. (B, D) Original magnification, ×400. Scale bars, 10 μm). Strong ANXA10 immunoreactivity is detected in primary OSCCs; normal oral tissues show almost negative immunostaining. (E) The state of ANXA10 protein expression in primary OSCCs (n = 100) and the normal counterparts. The ANXA10 IHC scores are calculated as follows: IHC score  = 1× (number of weakly stained cells in the field) +2× (number of moderately stained cells in the field) +3× (number of intensely stained cells in the field). The ANXA10 IHC scores for normal oral tissues and OSCCs ranged from 27.5 to 132.4 (median, 93.5) and 60.5 to 230.4 (median, 150.6), respectively. ANXA10 protein expression levels in OSCCs are significantly (*p<0.001, Mann-Whitney's *U* test) higher than in normal oral tissues.

Representative immunohistochemistry (IHC) results for ANXA10 protein in normal oral tissue and primary OSCC are shown in Figure 6A–D. The ANXA10 IHC scores of the cytoplasm of primary OSCCs were significantly (Figure 6E; *p<0.001) higher than in normal tissues, in which the IHC scores of normal oral tissues and OSCCs ranged from 27.5 to 132.4 (median, 93.5) and 60.5 to 230.4 (median, 150.6), respectively. Table 1 shows the correlations between the clinicopathologic characteristics of the patients with OSCC and the status of ANXA10 protein expression using the IHC scoring system. Among the clinical classifications, ANXA10-positive OSCCs were correlated with tumoral size (*p* = 0.027) and the TNM stage of the OSCCs (*p* = 0.041).

**Table 1:** Correlation between ANXA10 expression and parameters in OSCCs.

		Results of immunostaining No. patients		
Parameter	Total	ANXA10(−)	ANXA10(+)	*p-* value
Age at surgery				
<60	26	13 (50%)	13 (50%)	0.155
60–70	24	10 (42%)	14 (58%)	
70>	50	16 (32%)	34 (68%)	
Gender				
Male	64	26 (41%)	38 (59%)	0.713
Female	36	13 (37%)	23 (63%)	
T-primary tumoral size				
T1	5	2 (40%)	3 (60%)	0.027*
T2	66	32 (48%)	34 (52%)	
T3	13	3 (23%)	10 (77%)	
T4	16	2 (12%)	14 (88%)	
T1+T2	71	34 (48%)	37 (52%)	0.025*
T3+T4	29	5 (17%)	24 (83%)	
N-regional lymph node				
Negative	62	25 (40%)	37 (60%)	0.772
Positive	38	14 (37%)	24 (63%)	
TNM stage				
I	4	2 (50%)	2 (50%)	0.041*
II	45	22 (49%)	23 (51%)	
III	18	7 (39%)	11 (61%)	
IV	33	8 (24%)	25 (76%)	
Histopathological type				
Well-differentiated	59	19 (32%)	40 (68%)	0.177
Moderately differentiated	38	19 (50%)	19 (50%)	
Poorly differentiated	3	1 (33%)	2 (67%)	
Tumoral site				
Gingiva	49	19 (39%)	30 (61%)	0.843
Tongue	32	14 (44%)	18 (56%)	
Buccal mucosa	10	3 (30%)	7 (70%)	
Oral floor	9	3 (33%)	6 (67%)	
Total	100	39 (39%)	61(61%)	

ANXA10(-), down-regulated ANXA10; ANXA10(+), up-regulated ANXA10. *p<0.05.

## Discussion

In the current study, ANXA10 was frequently overexpressed in OSCC-derived cellular lines, and down-regulation resulted in decreased cellular proliferation by inactivation of ERK. The cell-cycle analysis also showed that G1 arrest occurred in ANXA10 knockdown cells through decreased CDKI (p21^Cip1^ and p27^Kip1^) expression. In addition, ANXA10 was frequently up-regulated in primary OSCCs and higher ANXA10 expression was associated with tumoral size (*p* = 0.027). These results indicated that ANXA10 is linked to regulation of the cell cycle in the G1 phase and plays an important role in tumoral progression in OSCCs.

Since oral carcinogenesis and other cancer types are thought to be multistep processes involving progressive disruption of epithelial cell proliferation, the mechanisms of cellular proliferation in tumoral cells are an important field of study for tumoral treatment and molecular cancer biology [30], [31]. Annexins, including ANXA10, have played important roles in tumoral development and progression [14]–[16], [18], [19]. However, no direct evidence has shown that ANXA10 is required for cellular proliferation. To determine whether ANXA10 function is relevant to OSCC progression, we performed functional studies using shRNA and found that suppression of ANXA10 significantly decreased cellular proliferation as a result of inhibited MAPK signaling pathway with down-regulation of pERK. The ERK/MAPK signaling pathway is related to many cellular processes, such as proliferation, differentiation, homeostasis, and cellular survival [17], [32]. Activation of the ERK/MAPK signaling pathway promotes abnormal cellular growth and tumorigenesis in multiple types of tumors. Our findings indicated that ANXA10 expression was relevant to phosphorylation of ERK and activation of the ERK/MAPK signaling pathway. The ERK/MAPK pathway is tightly controlled by mechanisms of extracellular signals. This includes the control of intracellular Ca^2+^ concentrations, thereby enabling Ca^2+^ to serve as a second messenger [33]. Ca^2+^-effector proteins can mediate cellular responses to changes in intracellular Ca^2+^ levels. Annexins, a family of such highly conserved Ca^2+^-regulated proteins, are characterized by their ability to bind to phospholipids in a Ca^2+^-dependent manner and most annexin functions are linked to their ability to interact with cellular membranes in a regulated manner [12], [13]. Based on these evidences combined with our results, we suggest that ANXA10 may affect intracellular Ca^2+^ homeostasis and directly or indirectly interacts with the activation of ERK/MAPK signaling pathways.

In addition to the MAPK signaling pathway, OSCC cells with shANXA10 revealed cell-cycle arrest at the G1 phase with up-regulation of p21^Cip1^ and p27^Kip1^ and down-regulation of cyclin D1 and cyclin E. The last two are also critical regulators of G1 progression and G1-S transition. Inhibiting their expression blocks G1-S transition in the cell cycle. The activities of the cyclin-CDK complexes are modulated by two types of CDKIs, Cip/Kip (p21^Cip1^and p27^Kip1^), which regulates cell-cycle progression. Members of the Cip/Kip family bind to cyclin-CDK complexes and inhibit their activities, which leads to G1 cell-cycle arrest. Cyclin D1, cyclin E, p21^Kip1^, and p27^Kip1^ levels are affected by multiple signaling pathways including the ERK/MAPK signaling pathway [25], [34]. Cyclin D1 and cyclin E are frequently up-regulated in human cancers [35], [36]. The mechanism of activating ERK in nonadherent fibroblasts has been reported to affect cyclin D1 [37]. In other reports, activation of ERK leads to up-regulation of cyclin D1 [38]. The CDKIs, p21^Kip1^ and p27^Kip1^, are frequently down-regulated in human cancers [39], [40]. The mechanism of ERK regulation of p21^Kip1^ and p27^Kip1^ remains unclear. ERK may directly phosphorylate p21^Kip1^ and p27^Kip1^ [41]–[43]. These data suggested that ANXA10 activates the MAPK signaling pathway by phosphorylating ERK and promotes the G1 cell cycle by down-regulating CDKIs in OSCC progression.

To date, the expression of ANXA10 in OSCC tissues and its correlation to clinicopathological features have not been elucidated. In our study, though the positive rates of ANXA10 in early-stage tumors were significantly low, the positive rates in advanced-tumors were significantly increased; this strongly implies that ANXA10 may play an important role in cancer progression. Given that ANXA10 overexpression may predict malignancy, patient stratification by ANXA10 status may provide a more personalized approach to human oral cancer therapy.

In conclusion, our results indicated that ANXA10 is overexpressed frequently in OSCCs and that this overexpression may be associated with tumoral progression by promoting cell-cycle progression in the G1 phase through activation of the ERK/MAPK signaling pathway, leading to decreased expression of CDKIs. While further studies are needed to study the interaction of ANXA10 and the ERK/MAPK signaling pathway, these data suggested that ANXA10 plays an important role in cellular proliferation and expression is likely to be a biomarker of progression and a potential therapeutic target for development of anticancer drugs in primary OSCCs.

## Materials and Methods

### Ethics Statement

The study protocol was approved by the Ethical Committee of Graduate School of Medicine, Chiba University (The approval number, 236) and was performed in accordance with the ethical standards laid down in the Declaration of Helsinki. Written informed consent was received from all patients.

### OSCC cellular lines and tissue samples

The human OSCC cellular lines (HSC-2, HSC-3, HSC-4, KON, Ho-1-u-1, and Ca9-22) were purchased from the Human Science Research Resources Bank, Osaka, Japan. Sa3 was kindly provided by Dr. S. Fujita at Wakayama Medical University, Wakayama, Japan [44]. Primary cultured HNOKs served as normal controls [45]–[49]. All cells were maintained in Dulbecco's modified Eagle's medium/F-12 HAM (Sigma, St. Louis, MO, USA) supplemented with 10% fetal bovine serum (Sigma) and 50 units/ml penicillin and streptomycin (Sigma) in a humidified 5% CO_2_/air atmosphere at 37°C.

One hundred pairs of primary OSCC samples and corresponding normal oral epithelial tissues were obtained during surgeries at Chiba University Hospital, Chiba, Japan. All patients provided informed consent for use of the protocol, which the institutional review board of Chiba University reviewed and approved. The resected tissues were divided into two parts; one was frozen immediately and stored at −80°C until RNA isolation, and the second was fixed in 20% buffered formaldehyde solution for pathologic diagnosis and IHC. Histopathological diagnosis of each tissue was performed according to the World Health Organization criteria by the Department of Pathology, Chiba University Hospital. Clinicopathological staging was determined according to the tumor-node-metastases classification of the International Union against Cancer. All OSCC samples were confirmed histologically and checked to ensure the presence of tumoral in more than 80% of specimens.

### Quantitative real-time reverse transcription-PCR

Total RNA was isolated using Trizol Reagent (Invitrogen, Carlsbad, CA, USA) according to the manufacturer's instructions. cDNA was generated from 5 µg of total RNA using Ready-To-Go You-Prime First-Strand Beads (GE Healthcare, Buckinghamshire, UK) and oligo (dT) primer (Hokkaido System Science, Sapporo, Japan) according to the manufacturer's instructions. qRT-PCR was performed to evaluate the expression level of *ANXA10* mRNA in the OSCC-derived cellular lines and HNOKs. The expression levels were determined using primers and probes that were designed using the Universal Probe Library (Roche Diagnostics, Mannheim, Germany) following the manufacturer's recommendations. The primer sequences used for qRT-PCR were: *ANXA10*, forward 5′- GCATCCATTATGGGATTGAAA-3′, reverse 5′- CAAAATGTTTTGTGGAGACTATGTG-3′, and universal probe #24. All qRT-PCR was performed using a LightCycler® 480 PCR system (Roche). Amplifications were initiated by a 10-minute pre-incubation at 95°C, followed by 45 cycles of 10 seconds at 95°C for template denaturation, 30 seconds at 55°C for primer annealing/extension and cooling for 30 seconds at 40°C. The transcript amount for the *ANXA10* was estimated from the respective standard curves and normalized to the *glyceraldehyde-3-phosphate dehydrogenase* (*GAPDH*) (forward 5′-CATCTCTGCCCCCTCTGCTGA-3′cell, reverse 5′-GGATGACCTTGCCCACAGCCT-3′, and universal probe #60) transcript amount determined in corresponding samples.

### Immunoblotting analysis

The cells were washed twice with cold phosphate-buffered saline (PBS) and centrifuged briefly. The cellular pellets were incubated at 4°C for 30 minutes in a lysis buffer (7 M urea, 2 M thiourea, 4% w/v CHAPS, and 10 mM Tris [pH 7.4]) with the proteinase inhibitor cocktail (Roche). The protein concentration was measured with a Bio-Rad Protein Assay (Bio-Rad Laboratories, Hercules, CA, USA). Protein extracts (20 µg) were separated by sodium dodecyl sulfate polyacrylamide gel electrophoresis in 4–12% gel, transferred to nitrocellulose membranes, and blocked for 1 hour at room temperature in Blocking One (Nacalai Tesque, Tokyo, Japan). The membranes were incubated with rabbit anti-ANXA10 polyclonal antibody (Abnova, Taipei, Taiwan), mouse anti-*α*-tubulin monoclonal antibody (Santa Cruz Biotechnology, Santa Cruz, CA. USA), mouse anti-ERK 1/2 monoclonal antibody (R&D Systems, Abingdon, UK), rabbit anti-phosphorylated ERK 1/2 monoclonal antibody (pERK, R&D systems), mouse anti-p21^Cip1^ monoclonal antibody, rabbit anti-p27^Kip1^ polyclonal antibody, mouse anti-Cyclin D1 monoclonal antibody (Cell Signaling Technology, Danvers, MA), mouse anti-Cyclin E monoclonal antibody (Santa Cruz Biotechnology), rabbit anti-CDK2 monoclonal antibody, mouse anti-CDK4 monoclonal antibody, or mouse anti-CDK6 monoclonal antibody (Cell Signaling Technology) for 4 hours at room temperature. The membrane was washed with 0.1% Tween-20 in Tris-buffered saline, incubated with secondary antibody and coupled to horseradish peroxidase-conjugated anti-rabbit or anti-mouse IgG (Promega, Madison, WI, USA) for 1 hour at room temperature. The proteins were detected by SuperSignal Chemiluminescent substrate (Thermo, Waltham, MA, USA). Finally, the immunoblotting analysis results were visualized by exposing the membrane to a cooled CCD camera system (ATTO, Tokyo, Japan). Signal intensities were quantitated using the CS Analyzer version 3.0 (ATTO).

### Transfection with shRNA plasmid

The OSCC cellular lines, Sa3 and Ho-1-u-1, in which ANXA10 protein expression was higher than in the other cellular lines, were stably transfected with the ANXA10 shRNA (shANXA10) or the control shRNA (shMock) (Santa Cruz Biotechnology) using Lipofectamine LTX and Plus Reagents (Invitrogen). The stable transfectants were isolated by the culture medium containing 2 mg/ml Puromycin (Invitrogen). Two to 3 weeks after transfection, viable colonies were picked up and transferred to new dishes. shANXA10- and shMock-transfected cells were used for further experiments.

### Proliferation assays

To investigate the effect of ANXA10 knockdown on cellular proliferation, shANXA10- and shMock-transfected cells were seeded in six-well plates at a density of 1×10^4^ viable cells/well. At the indicated time points, the cells were trypsinized and counted in triplicate using a hemocytometer.

### Cell-cycle analysis

In order to synchronize cells at the G0/G1 or G2/M transition, the cells were deprived of serum for 48 hours or treated with 200 ng/ml nocodazole (Sigma) for 20 hours [50], [51]. To determine the cell-cycle distribution, the cells were harvested, washed with PBS, and probed with CycleTEST Plus DNA reagent kit (Becton-Dickinson, San Jose, CA, USA), according to the manufacturer's protocol. Briefly, the cells concentrated to 5×10^5^ cells/ml were centrifuged at 400×g for 5 minutes at room temperature. We added 250 ml of Solution A (trypsin buffer), and incubated the mixture for 10 minutes at room temperature. We then added 200 ml of Solution B (trypsin inhibitor and RNase in a buffer), and incubated the mixture for 10 minutes at room temperature. Finally, we added 200 ml of Solution C (propidium iodide stain solution), and incubated the mixture for 10 minutes in the dark on ice. Flow cytometric determination of DNA content was analyzed by FACScalibur (Becton-Dickinson). The fractions of the cells in the G0–G1, S, and G2–M phases were analyzed using FlowJo software (Tree Star, Ashland, OR, USA).

### Immunohistochemistry

IHC was performed on 4-µm sections of paraffin-embedded specimens using rabbit anti-ANXA10 polyclonal antibody (Abnova). Briefly, after deparaffinization and hydration, the endogenous peroxidase activity was quenched by a 30-minute incubation in a mixture of 0.3% hydrogen peroxide solution in 100% methanol. The sections were blocked for 2 hours at room temperature with 1.5% blocking serum (Santa Cruz Biotechnology) in PBS before reacting with anti-ANXA10 antibody (1∶1000 dilution) at 4°C in a moist chamber overnight. Upon incubation with the primary antibody, the specimens were washed three times in PBS and treated with Histofine Simplestain Max-PO (G) (Nichirei, Tokyo, Japan) followed by color development in 3,3′-diaminobenzidine tetrahydrochloride (DAKO, Carpinteria, CA, USA). Finally, the slides were counterstained lightly with hematoxylin, dehydrated with ethanol, cleaned with xylene, and mounted. Nonspecific binding of the antibody to proteins other than the antigen sometimes occurred. As a negative control, triplicate sections were immunostained without exposure to primary antibody, which confirmed the staining specificity. To quantify the state of ANXA10 protein expression in those components, we used IHC scoring systems described previously [48], [52]–[55]. The intensity of the ANXA10 immunoreaction was scored as follows: 1+, weak; 2+, moderate; and 3+, intense. The cellular numbers and the staining intensity then were multiplied to produce the ANXA10 IHC score. Cases with an ANXA10 IHC score exceeding 132.0 (+3 standard deviation (SD) score for normal tissue) were defined as ANXA10-positive. The ±3 SD cutoff, which statistically is just 0.2% of the measurement and is expected to fall outside this range, was used because it was unlikely to be affected by a random experimental error produced by sample manipulation [56]. Two independent pathologists, neither of whom had knowledge of the patients' clinical status, made these judgments.

### Statistical analysis

The significance of the ANXA10 expression levels was evaluated using Fisher's exact test or the Mann-Whitney U test. *P*<0.05 was considered statistically significant. The data are expressed as the mean ± standard error of the mean (SEM).
